# Effect of retirement on COVID-19 vaccination in Europe: a quasi-experimental study

**DOI:** 10.1038/s41541-025-01290-y

**Published:** 2025-11-20

**Authors:** Ilias Kyriopoulos, Sofia Tsarsitalidou, Elias Mossialos

**Affiliations:** 1 Department of Health Policy, London School of Economics and Political Science, Cowdray House, Houghton Street, London, UK; 2 LSE Health, London School of Economics and Political Science, Cowdray House, Houghton Street, London, UK; 3 Department of Accounting and Finance, University of Ioannina, Preveza, Greece

**Keywords:** Public health, Health care, Risk factors

## Abstract

This study investigates how the transition to retirement influenced the uptake of COVID-19 vaccination in Europe. We employed a quasi-experimental regression discontinuity design to assess the impact of retirement on COVID-19 vaccination, accounting for unobserved confounding and controlling for factors such as subjective health status, age, vaccination for other diseases, medication use, cancer diagnosis, and internet use. We used individual-level data from the Corona Surveys of the Survey of Health, Ageing, and Retirement in Europe (SHARE), conducted in 2020 and 2021. Our outcomes were binary indicators capturing whether the respondents were either vaccinated or expressed the intention to get vaccinated. The independent variable of interest was retirement status, which is instrumented using age eligibility for pension benefits as discontinuous policy threshold in a fuzzy regression discontinuity design. Estimates indicated that retirement was associated with a decline in COVID-19 vaccination uptake. Specifically, retirees were less inclined to get vaccinated compared to individuals of similar age who had not yet retired. The estimates remained robust to different model specifications and falsification tests. Changes in the frequency of social contacts following retirement could offer a plausible explanation for the observed relationship. Given that retirees may have fewer social interactions and, as a result, reduced exposure to potential health risks, they might have viewed social isolation as a potential substitute for COVID-19 vaccination.

## Introduction

Retirement status poses challenges across multiple policy domains, ranging from fiscal sustainability of the pension system to behavioral changes and implications for individuals entering retirement. Focusing on the latter, the transition to retirement is considered one of the most significant life events and has the potential to result in substantial changes in an individual’s behavior and daily life. Retirement can alter individual preferences due to a range of factors, including increased leisure time, the potential emergence of myopic or time-inconsistent behaviors, reduced income and financial liquidity, as well as changes in daily routines and social networks^1,2^.

The impact of retirement on health and health behaviours has been extensively studied (see the Supplementary Material for a literature review)^3–6^. However, this body of work focuses on sustained and ongoing behaviors, such as physical exercise, smoking, and alcohol consumption^4,7–9^. There is lack of evidence on how retirement affects COVID-19 vaccination, which is a more episodic health behavior involving complex decision-marking during a public health crisis. Decisions about vaccine acceptance are multifaceted and influenced by various factors, including risk perception, vaccine confidence (i.e., trust in and concerns about safety, efficacy, and effectiveness), adherence to social norms, recommendations from health providers, and practical considerations, such as access to information, vaccine availability, ease of access, and satisfaction with vaccination services^10,11^. Retirement may be associated with several of these factors. For instance, it may change access to health care and information, reshape social networks, or affect perceived risk—all of which may influence vaccination decisions^2,12^.

Determining the potential impact of retirement on vaccination is not straightforward. There might be several reasons why retirement can increase willingness to get vaccinated. The lower opportunity cost of time may provide retirees with more time to invest in preventive health measures. Meanwhile, enhanced altruistic and prosocial behaviors upon retirement^13^ may extend to a greater willingness to protect the wider community through vaccination. However, certain factors may reduce vaccine uptake. A tendency toward smaller social networks and less frequent social interactions following retirement^2^ may influence individuals’ risk perception. Reduced contact with potential virus carriers, limited exposure to information, and fewer peer-driven social influences^14^—such as those shaping social identity and promoting collective behaviour—could all play a role in this altered risk perception^15^. Retirement has been also associated with cognitive decline^16^ which, in turn, was found to predict vaccine uptake during the COVID-19 pandemic^17^. Retirement may also bring about feelings of loneliness, obsolescence, and a diminished sense of purpose^7^, all of which have been linked to a decreased willingness to vaccinate against COVID-19. Furthermore, retired individuals are no longer subject to potential workplace restrictions, such as the need for vaccination certification during the pandemic, making it easier for those who are hesitant to avoid vaccination. While price may be considered as a determinant of vaccine uptake, we do not expect it to be a major factor in this case, as COVID-19 vaccines were provided free of charge. However, retirees—often living on reduced or fixed incomes—may still face income-related access barriers, such as transportation costs or challenges navigating appointment systems, which could hinder vaccine uptake despite the absence of direct costs.

Our study focused on COVID-19 vaccination for several reasons that extend beyond its critical role in public health, including the prevention of severe symptoms, hospitalization, and death. COVID-19 vaccines were developed, produced, and distributed rapidly during a major public health crisis, and were not initially part of routine immunization schedules. Unlike other vaccines—typically administered periodically or targeted to specific age groups or risk profiles—COVID-19 vaccination efforts involved mass national campaigns, centralized registration systems, and widespread public communication. This unique delivery context, combined with intense media attention and political discourse, made COVID-19 vaccines far more visible and socially salient^18^. Retirees may have responded differently to this context compared to other, more routine vaccinations, either due to reduced exposure to workplace mandates or differential exposure to pandemic-specific misinformation and public anxiety. Moreover, the pandemic brought vaccine hesitancy and the broader determinants of vaccine uptake to the forefront of the public health agenda. Already unstable public sentiment regarding vaccines became even more pronounced during the COVID-19 pandemic^19,20^. Taken together, these contextual distinctions underscore why retirement may influence COVID-19 vaccination behavior in ways that are not necessarily generalizable to other adult vaccines, thereby justifying our focused analysis.

COVID-19 vaccines were centrally authorized by the European Medicines Agency, but individual countries retained control over prioritization, distribution, and mandates. Although European Union (EU) countries provided vaccines free of charge to the public, they differed in their rollout strategies, booster eligibility criteria, and data reporting practices. Nevertheless, this divergence in vaccination policies was less pronounced compared to that observed in non-pharmaceutical interventions^21^. Most countries prioritized older adults and vulnerable populations during the initial phases of the vaccination campaign. Despite policy differences, all countries ultimately extended vaccine access to the general adult population, thereby minimizing financial and eligibility-related barriers. National health systems across Europe generally ensured full coverage of vaccination costs, with administration carried out through centralized centres, outpatient clinics, and community outreach efforts.

We investigated how the transition to retirement is linked with the uptake of COVID-19 vaccination. While several studies have explored the factors influencing vaccine uptake, most of this evidence is primarily correlational and may lack strong internal validity. In this context, there are several unobservable time-varying factors that influence both an individual’s decisions to retire and their likelihood of getting vaccinated against COVID-19. For example, evolving risk perceptions related to the pandemic—shaped by personal experiences or media exposure—may influence both the perceived need to leave the workforce and attitudes toward vaccination. To address this challenge, we employed a fuzzy regression discontinuity design (RDD) that leveraged the discontinuities arising from eligibility rules for ERA across different countries.

This paper contributes to four distinct areas of research. First, our study adds to the expanding body of evidence concerning the effects of retirement on health behaviors. Existing literature on this topic has produced inconclusive findings, often focusing more on health outcomes than preventive behaviors. Second, our research contributes to the broader literature on the determinants of COVID-19 vaccination uptake. While a substantial body of work has identified various socioeconomic, demographic, and health-related factors associated with COVID-19 vaccination, we extend this literature by using a quasi-experimental approach to investigate the impact of retirement on vaccination. Third, we tackle concerns related to potential endogeneity and provide robust evidence regarding the influence of retirement on vaccination behavior, focusing specifically on COVID-19 vaccines. To the best of our knowledge, this study is the first of its kind to do so. Moving beyond routine vaccinations, we particularly examined how retirement influences vaccine uptake during high-salience public health campaigns such as those launched in response to the COVID-19 pandemic. We thus explored how major life-course transitions shape vaccination behaviour in times of public health crises. Fourth, we empirically test potential mechanisms that help explain the connection between retirement and vaccination. In particular, we shedded light on the role of social contact frequency, which may be a key mechanism linking retirement to vaccination during a pandemic. Social contacts affect exposure risk, access to information, and social norms around vaccination. Since retirement often alters daily social routines, examining contact frequency provides insight into how life transitions influence health behaviours during a crisis. Top of Form

## Results

### Main results

Table 1 provides the descriptive statistics for the variables included in our analysis. The full sample comprises 47,407 individuals, of whom 71.7% are retired. Our analysis focused on observations near the threshold, selected using an optimal bandwidth approach^22,23^. Consequently, our working sample consisted of 11,446 individuals, with 37.4% classified as retired. The mean age of the respondents was 62.3 years (standard deviation: 3.03). Out of the 6761 individuals eligible for early retirement within the study’s bandwidth, 3516 were retired.

**Table 1:** Descriptive statistics (baseline bandwidth)

Variable	Frequency (%)	Number of observations
Vaccinated	83.9	9675
Other Vaccine	19.4	2229
Male	43.6	4939
Female	56.4	6382
Subjective health		
Excellent	6.7	773
Very good	22.1	2551
Good	42.8	4940
Fair	22.9	2643
Poor	5.5	635
On prescription drugs	65.7	7580
Cancer	4.4	507
Internet use	74.9	8640
Contact freq. family		
Never	8.0	624
Rarely	28.7	2239
Sometimes	27.0	2106
Often	24.1	1880
Daily	12.1	944
Contact freq. friends		
Never	9.7	1114
Rarely	29.3	3364
Sometimes	19.2	2204
Often	23.1	2652
Daily	18.7	2147

Table 2 contains the results regarding the relationship between retirement status and COVID-19 vaccination, with each column corresponding to a different estimation. The estimates of our baseline model—which draws on the nonparametric approach with optimal bandwidth selection^22,23^—are presented in Column 1. The results suggest that retired individuals have had a lower probability of getting vaccinated. Column 2 presents the estimates of the nonparametric (baseline) estimation after changing the outcome variable. In Column 2, we only focused on actual vaccination, and excluded those willing to get vaccinated in the future (potentially due to lack of availability in their country at the time of the survey) or already scheduled their vaccination. Therefore, the only difference with Column 1 is the change in outcome variable, with the results holding.

**Table 2:** Retirement and COVID-19 vaccination (non-parametric estimates)

	(1)	(2)	(3)	(4)	(5)
	Nonparametric model	Nonparametric model using alternative dep. variable	Excluding permanently sick or disabled	Excluding individuals without chronic diseases	Excluding homemaker
Second Stage Estimates					
Retirement status	−0.659**	−0.651**	−0.790**	−0.428***	−0.448*
	(−2.35) (−1.93)	(−2.00) (−1.65)	(−2.39) (−1.96)	(−2.59) (−2.15)	(−1.78) (−1.46)
	[−1.20, −0.109]	[−1.28, −0.013]	[−1.43, −0.14]	[−0.751, −0.104]	[−0.93, 0.04]
First Stage					
Estimates	0.042***	0.046***	0.030*	0.055***	0.047***
Eligibility for ER	(2.56) [0.010, 0.075]	(2.60) [0.011, 0.080]	(1.77) [−0.003, 0.063]	(3.31) [0.022, 0.088]	(2.61) [0.011, 0.082]
Observations	11446	10343	11304	11689	10434
Covariates	Yes	Yes	Yes	Yes	Yes
Country dummies	Yes	Yes	Yes	Yes	Yes
Bandwidth	49.634	44.025	51.61	63.63	63.63
Polynomial Order	1	1	1	1	1

The results are local polynomial regression discontinuity (RD) estimates using the optimal bandwidth selection procedure^22,23^. Two different methods (bias-corrected and robust estimator) for computing heteroskedasticity robust standard errors apply. The covariates used are subjective health status, age, vaccination status for other viruses, use of medicine, having cancer and the use of the internet since the start of the pandemic. Bias corrected t-statistics in the first parenthesis and robust t-statistic in the second. The 95% confidence interval of the bias-corrected estimator is presented in square brackets). *, **, *** indicate significant at the 10%, 5%, 1% level, respectively.

We also wanted to ensure that specific individual characteristics did not drive the negative relationship between retirement and COVID-19 vaccination. Column 3 excludes from our sample individuals with long-term illnesses. This group of individuals might be in receipt of some form of pension despite being younger than the early retirement threshold and, in addition, might be at a higher risk of severe COVID-19 infection, thus increasing their probability of getting vaccinated. Importantly, our results remain consistent even after removal of this group from the analysis. Column 4 excludes individuals who did not have chronic diseases from the analysis. This subgroup could be considered relatively healthier than those with chronic conditions. The rationale for this exclusion was to assess whether the results were driven by a relatively healthier population group, who might have a lower risk of severe COVID-19 infection and potentially less incentive to get vaccination. Even after excluding this group, our results remain consistent. Column 5 excludes homemakers from our analysis, as these individuals often spend more time at home and may have less frequent social interactions outside the household. Their reduced social interaction might have made it easier for them to avoid potential exposure to the virus. Even after excluding homemakers from the sample, our results remained unchanged.

In Table S1 in the Supplementary Material, we presented the results of the McCrary test, which show that there is no evidence of manipulation of the assignment variable. Additionally, as shown in the Supplementary Material Table S2, the results are consistent and robust across alternative specifications. Column 1 shows the results of the 2SLS estimates derived from the parametric model corresponding to Eq. (1) and (2), using a bandwidth of four years around the threshold. The results again suggest that retired individuals have had a lower probability of getting vaccinated. We also estimated a similar model using a three-year bandwidth and found similar results for the direction and size of the coefficient (see column 2). Column 3 presents the 2SLS estimates following adoption of a quadratic functional form, with results similar to the previous specifications. Column 4 shows the estimates from the baseline nonparametric model, controlling for country dummies but excluding other covariates. The findings remain robust. Apart from the second-stage results, the first-stage results are consistently significant and positive, implying that individuals crossing the early retirement threshold had a greater probability of retiring.

In the Supplementary Table S3, we also explored some potential mechanisms driving this relationship, by testing potential discontinuities in variables related to the frequency of social contact. The estimates presented in Table S3 reveal that retirement is associated with an increase in the frequency of social contact with close family members, such as children or grandchildren. However, it simultaneously results in a decrease in social contact with neighbors, friends, or colleagues. These findings suggest that retired individuals may have been inclined to avoid vaccination because they believed they could protect themselves by reducing their interactions with certain social groups that they perceived as posing a higher risk of COVID-19 transmission. Essentially, not going to work or meeting friends less frequently reduced their exposure to potential health risks, which in turn may have been seen as a substitute for getting vaccinated against COVID-19.

### Supplementary analyses

To further test the robustness of our baseline findings, we performed a Jackknife analysis^24^. This approach is based on estimating the nonparametric baseline equation 26 times and excluding one cross sectional unit (i.e., country) in each replication. Table S4 reports the estimated coefficients along with the excluded countries. Comparing these coefficients with those presented in Table 2, we can conclude that our results are robust to the exclusion of countries. We also estimated the baseline equation after including and excluding covariates, as well as for subgroups of individuals without certain health conditions—specifically cancer, chronic lung disease, high blood pressure, diabetes, and heart attack. The results, presented in Tables S2, S5 and S6 in the Online Supplementary Material, remain robust.

To further confirm the validity of our findings, we examined whether there was a discontinuity of the covariates used at the early retirement threshold. Further, we ruled out the possibility that our main finding was driven by other variables associated with COVID-19 vaccination (i.e., that there was no jump in the probability of vaccination, but rather in another variable that affected it). For this reason, we performed a fuzzy regression discontinuity estimation to examine whether relevant covariates exhibited a jump at the threshold. The results indicate that the variables that potentially affected vaccination against COVID-19 did not change discontinuously at the threshold (see Table S7). In any case, we also included these covariates in our main specification to further ensure the validity of our results. We also tested whether, in a local neighborhood near the cutoff, the number of observations below the cutoff differed from those above it. We provided a formal test in which the null hypothesis suggested no difference in the density of treated and control observations at the cutoff *(*$T=1.2879,{p\_value}0.197$*)*. In order to consider household dynamics, we also included both own and partner’s retirement in the model. As shown in Table S8 in the Online Supplementary Material, our results remain robust to this adjustment.

As falsification tests, Table 3 reproduces the results of our baseline specification, using placebo early retirement age (ERA) thresholds to test for anticipation effects of retirement on COVID-19 vaccination. We present the results using the values -24, -12, 12, and 24 months as cutoff points in the age-centered variable. As expected, the results were not statistically significant.

**Table 3:** Placebo tests

	(1)	(2)	(3)	(4)
	Placebo -2 years	Placebo -1 year	Placebo 1 year	Placebo 2 years
Second Stage Estimates				
Retirement	1.712	0.173	−0.145	0.0239
status	(0.52) (0.42) [−4.71, 8.13]	(0.38) (0.31) [−0.72, 1.06]	(−0.50) (−0.46) [−0.71, 0.42]	(0.09) (0.08) [−0.49, 0.54]
First Stage				
Estimates	0.017	0.04	0.02	0.03
Eligibility for ER	(−1.02) [−0.050, 0.015]	(2.18) [0.004, 0.768]	(1.45) [−0.008, 0.058]	(2.17) [0.003, 0.073]
Observations	6890	7644	12067	11280
Covariates	Yes	Yes	Yes	Yes
Country dummies	Yes	Yes	Yes	Yes
Bandwidth	35.09	35.97	48.02	42.75
Polynomial Order	1	1	1	1

All results are local polynomial regression discontinuity (RD) estimates using the optimal bandwidth selection procedure^22,23^. Two different methods (bias-corrected and robust estimator) in computing standard errors apply. Bias corrected t-statistics in the first parenthesis and robust t-statistic in the second. The 95% confidence interval of the bias-corrected estimator is presented in square brackets). *, **, *** indicate significant at the 10%, 5%, 1% level, respectively.

Table 4 further presents the results of a ‘donut hole’ approach we took to test whether the results were sensitive to the observations near the cutoff. We did this to exclude units that were more likely to have engaged in manipulation. To this end, we dropped individuals aged 1, 3 and 6 months respectively above or below the ERA threshold, using the optimal bandwidth calculated in column 1 of Table 2. The estimates show that the observations closest to the threshold did not drive our results. Overall, the robustness checks confirm our baseline results and lend support to the hypothesis that retirement affected vaccination uptake against COVID-19.

**Table 4:** Donut Hole’ RDD to test sensitivity to the observations near the cutoff

	(1)	(2)	(3)
	Donut hole (1 month)	Donut hole (3 months)	Donut hole (6 months)
Second Stage Estimates			
Retirement	−0.891**	−0.530*	−0.048*
status	(−2.33) (−1.49) [−1.64, −0.13]	(−1.77) (−1.15) [−1.11, 0.56]	(−1.71) (−1.14) [−1.03, 0.70]
First Stage			
Estimates	0.044***	0.06***	0.06***
Eligibility for ER	(2.57) [0.010, 0.078]	(3.59) [−0.028, 0.096]	(3.76) [0.031, 0.098]
Observations	11202	11192	11221
Covariates	Yes	Yes	Yes
Country dummies	Yes	Yes	Yes
Bandwidth	46.634	46.634	46.634
Polynomial Order	1	1	1

All results are local polynomial regression discontinuity (RD) estimates.22,23 Two different methods (bias-corrected and robust estimator) in computing heteroskedasticity robust standard errors apply. Bias corrected t-statistics in the first parenthesis and robust t-statistic in the second. The 95% confidence interval of the bias-corrected estimator is presented in square brackets). *, **, *** indicate significant at the 10%, 5%, 1% level, respectively.

## Discussion

In this study, our primary inquiry was the association between retirement and COVID-19 vaccination. Using survey data and a regression discontinuity design, we exploited the exogenous variation in early retirement eligibility rules across countries to address potential endogeneity. The key assumption underpinning our approach was that the rate of COVID-19 vaccination would be similar around the early retirement threshold if the respondent had not retired. In other words, we used this threshold in a quasi-experimental setting to examine the link between retirement and COVID-19 vaccination rates. Having addressed concerns related to endogeneity, our analysis revealed a pattern: retirement was associated with a decreased likelihood of COVID-19 vaccination. Retirees were less inclined to get vaccinated compared to individuals of similar age who were still in the workforce. This aligns with a body of research suggesting that retirement may lead to a decrease in healthy behaviors^9,25^. Our estimates hold up well under various approaches and model specifications, strengthening the robustness of our findings.

We also provided evidence suggesting that changes in the frequency of social contacts following retirement may offer a plausible explanation for the observed relationship, especially considering that the size of social network has been found to be associated with COVID-19 vaccination^15,26,27^. Specifically, it appears that retirement was associated with a reduction in social interactions with neighbors, friends, or colleagues. This finding is consistent with previous research, which has shown that the size of one’s social network tends to decrease following retirement^2^. Fewer social interactions and, as a result, reduced exposure to potential health risks, may have led retirees to view social isolation as a potential substitute for COVID-19 vaccination. Moreover, an individual’s likelihood of getting immunized was often influenced by their perception of the risk of infection^28^, which is likely to be lower for retirees due to their absence of work-related commitments or their tendency to move to less densely populated areas.

From a behavioural science perspective, vaccination decision is driven by weighting the perceived risks of getting vaccinated with the risks of contracting the disease^29,30^. COVID-19 generates positive externalities; when more people are vaccinated, the overall risk of infection decreases, potentially encouraging some individuals to free-ride on the vaccination efforts of others due to rational self-interest. Certain groups—including retirees—may have incentives towards free-riding, as they may experience reduced daily social interactions or diminished workplace exposure, subsequently lowering their perceived risk of contracting COVID-19. Consequently, they might feel safer relying on the immunity established by others without bearing the perceived risks of vaccination themselves^30^. This underscores how retirement status can influence the propensity to engage in free-riding behaviours in the context of COVID-19 vaccination.

While our analysis specifically focused on COVID-19 vaccination, it is important to recognize that the factors influencing uptake and the attitudes toward other adult vaccines may differ substantially^31^. COVID-19 vaccines were uniquely deployed through rapid, large-scale national campaigns, frequently involving centralized, digital registration processes and extensive public messaging that heightened their visibility and urgency^18^. By contrast, uptake of other adult vaccines is generally determined by routine interactions with primary care providers, periodic recommendations, resulting in different barriers and facilitators for vaccination. Additionally, COVID-19 vaccine uptake was significantly influenced by intense media coverage and pandemic-specific misinformation^32^, factors less prominent for routine vaccinations. Retirement could also differentially affect vaccine uptake: retirees are no longer subject to workplace mandates or direct peer influences related to vaccination, factors that were particularly salient during the COVID-19 pandemic. Thus, while our study sheds light on the relationship between retirement and COVID-19 vaccination, it also highlights the necessity for future research exploring how retirement might influence decisions for other vaccines, each embedded within different institutional, informational, and behavioural contexts.

This study has some limitations. First, we were unable to offer insights into the long-term impact of retirement on vaccination due to methodological constraints and data limitations. While we recognize the importance of understanding post-retirement dynamics, our empirical approach did not permit us to fully explore these dynamics, as using observations far from the pension eligibility threshold could have introduced bias. Our focus was primarily on establishing relationships through the variation around the threshold. Second, it is important to acknowledge that at the time of the survey, the availability and accessibility of COVID-19 vaccines varied by country. Some individuals may have wanted to get vaccinated but were unable to do so due to limitations in vaccine availability and distribution in their respective countries. These variations across countries could have influenced the observed vaccination patterns. However, we took steps to mitigate the impact of variations in vaccine availability and accessibility across countries by creating a dummy variable that took the value of 1 if an individual was vaccinated or expressed a willingness to get vaccinated, and we conducted several checks to ensure the robustness of the results. We also performed additional analyses focusing on actual vaccination and without considering willingness to get vaccinated, with our findings remaining consistent. Third, due to limitations in data availability, we were unable to explore additional mechanisms that could provide a more comprehensive explanation of our findings, such as differences to exposure to information, types of information sources, transportation barriers or health consciousness. This represents a potential avenue for future research. Fourth, changes in vaccination behavior might be explained by potential transitions to other forms of employment after passing the ERA threshold. We cannot test such a hypothesis due to data availability restrictions. Nonetheless, this does not threaten the validity of our results, as we estimate the Local Average Treatment Effect for compliers (i.e., those who passed the ERA threshold and retired due to eligibility for old-age pension)^8^. With respect to generalizability, it is important to note that RDD estimates are internally valid in the sense that they address endogeneity concerns. However, we refrain from making claims for broader populations beyond the one for which we have estimated the relevant Local Average Treatment Effect. Fifth, vaccination status was based on self-reported data, which may be subject to recall bias. Last, due to data limitations, we were unable to test how retirement influences the number of vaccine doses received or the completion of booster vaccinations.

Despite the limitations mentioned, this study offers robust evidence regarding the impact of retirement on COVID-19 vaccination rates and provides credible mechanisms to elucidate this connection. Furthermore, the study carries implications for public policy. Firstly, in an era marked by mounting concerns about potential future global health crises, it is important to comprehend the causal drivers of vaccination uptake. This knowledge can inform the design of prevention policies and strategies for pandemic preparedness and response^33^. Secondly, our findings underscore the necessity for targeted public health and vaccination campaigns aimed at retirees. Given that retirees exhibit a lower likelihood of getting vaccinated compared to individuals of similar age still in the workforce, tailoring interventions to specific population groups could be important for increasing vaccination rates.

## Methods

### Data sources and outcomes

Our analysis employed data from the Corona Surveys 1 and 2, conducted in 2020 and 2021, as part of the Survey of Health, Ageing, and Retirement in Europe (SHARE). SHARE is a cross-national survey that collects data on individuals aged 50 or older, covering a wide range of domains including physical and mental health, cognition, health behaviors, social network, and socio-demographic characteristics. The following countries were included: Austria, Belgium, Croatia, Cyprus, Czech Republic, Denmark, Estonia, Finland, France, Germany, Greece, Israel, Italy, Latvia, Lithuania, Luxembourg, Malta, Netherlands, Poland, Portugal, Romania, Slovakia, Slovenia, Spain, Sweden, and Switzerland. The data for Corona Survey 1 and 2 were collected between June and August 2020 and June and August 2021, respectively, through computer-assisted telephone interviews^34^. Earlier studies examining the impact of retirement on health and health behaviors have also used SHARE data^8,35^.

Our primary variable of interest was vaccination against COVID-19, which we operationalized as a binary variable taking the value of 1 if a respondent was either vaccinated or expressed the intention to get vaccinated, and 0 otherwise. This approach allowed us to account for countries in which the vaccine was not yet available during the interview period. Given that the SHARE Corona Survey was conducted at different periods in each country, we used a dummy if a person was either vaccinated or wanted to get vaccinated. We did this to include individuals who were not eligible for vaccination but were willing to get vaccinated if the opportunity arose during the survey period. To ensure the robustness of our estimations, we also validated our findings using a binary variable based on actual vaccination status, without considering intentions to get vaccinated. We did so on the understanding that willingness to vaccinate might be susceptible to social norm bias (i.e., interviewer effect). In such cases, respondents might overstate their willingness to vaccinate in order to conform to perceived social norms or to appear favorable in the eyes of the interviewer. Further details regarding the measurement of COVID-19 vaccination variables are provided in Table S9 in the Online Supplementary Material. Furthermore, a dummy variable representing retirement status was created based on a question concerning current employment status.

### Study design

This study aimed to investigate the impact of retirement on COVID-19 vaccination while mitigating potential endogeneity issues. In this context, we needed to address two primary sources of endogeneity. The first relates to omitted variable bias, given that a simple correlational estimate would have failed to effectively account for unobservables that might influence both vaccination against COVID-19 and retirement decision, such as personality traits or subjective life expectancy. The second arises from the endogeneity of retirement decision and subsequent reverse causality. It is possible that individuals who were either compelled or strongly encouraged by their employers to get vaccinated might opt to retire to avoid vaccination, thus introducing reverse causality into the analysis.

Given that standard regression models would produce biased results, we employed a fuzzy RDD using individuals’ ages and early retirement eligibility set by each country, derived from the Organisation for Economic Co-operation and Development (OECD) database, to construct the retirement threshold (summarized in Table S10 in the Online Supplementary Material). Additionally, we also used sex-specific ERA thresholds. Our approach was consistent with previous studies that have used the ERA threshold as the basis of this identification strategy^8,9,13,36–38^.

In RDD, treatment assignment is determined by a predefined threshold on a forcing variable (i.e., age). In other words, an exogenous policy rule influences the probability of whether an individual receives the treatment. Depending on this rule, individuals are assigned to treatment or control groups based on whether they fall below or above a specific threshold in the forcing variable.

Under some assumptions, a discontinuity in the outcome variable at the threshold of the forcing variable can be interpreted as the effect of the treatment. In particular, the key assumption underpinning our empirical strategy is that the rate of COVID-19 vaccination would have evolved smoothly around the early retirement eligibility threshold if individuals had not retired. This assumption implies that individuals just below and just above the cutoff are similar in both observable and unobservable characteristics, except for their retirement status. Under this condition, any discontinuity in vaccination uptake at the threshold can be attributed to the effect of retirement, rather than other confounding factors. This is the standard identifying assumption in a fuzzy RDD, where the running variable (age) determines the probability of treatment (retirement). We support the plausibility of this assumption by conducting several robustness checks, including tests for the continuity of predetermined covariates around the threshold and sensitivity analyses with alternative bandwidths.

Additionally, one of the preconditions for the validity of the approach is that individuals cannot precisely manipulate the forcing variable^39^. In other words, if individuals can deliberately manipulate this variable to their advantage, the value of the forcing variable near the threshold may not accurately or randomly assign them into the treatment group, which could undermine the validity of the RDD analysis.

In our setting, we exploited information from the policy rules for ERA thresholds across countries. Our analysis thus drew on a fuzzy regression discontinuity design that exploited the discontinuities in the probability of retirement. We expected that the probability of retirement would increase discontinuously at the threshold for ERA. In particular, the ERA policy rules are not deterministic in nature, in the sense that some individuals who pass the ERA do not retire and others may exit the labour market earlier. Being older than the ERA does not necessarily imply retirement, as transitioning to retirement after reaching the ERA is not mandatory in any of the countries. In this case, the policy rule did not perfectly define treatment status, so there was imperfect compliance, with the probability of treatment being discontinuous at the threshold rather than simply switching from 0 to 1^40^.

Since retirement is affected by the eligibility rule, we used the information provided by SHARE to calculate the respondent’s age at the year of interview and then calculated the distance from ERA. The running variable was calculated in months and constructed as ${X}_{i,c}\,$= ${ag}{e}_{i}-{earlyag}{e}_{c}$, with $c$ corresponding to the different ERA thresholds across countries. Positive values of the running variable indicated early retirement eligibility since the individual was older than the ERA threshold set by their country.

A fuzzy RDD can be interpreted as a case of instrumental variables approach, closely resembling a two-stage lease squares (2SLS) model. In this setup, reaching the ERA induces a discontinuous change in the probability of retirement. The dummy variable taking the value of 1 when someone crosses the age threshold thus serves as an instrument for actual retirement status. In the first stage, crossing the retirement age predicts the likelihood of retirement. In the second stage, the predicted retirement status is used to estimate its impact on vaccination. This allows us to estimate the local average treatment effect (LATE) for individuals who retire after reaching the age threshold (i.e., the compliers).

We first estimated parametric model of the following form:

1 $${D}_{{ic}}={\pi }_{0}+{\pi }_{1}{X}_{{ic}}+{\pi }_{2}{T}_{{ic}}+{\pi }_{3}{Z}_{{ic}}+{\lambda }_{i}+{v}_{{ic}}$$

2 $${Y}_{{ic}}={\beta }_{0}+{\beta }_{1}{\hat{D}}_{{ic}}+{\beta }_{2}{X}_{{ic}}+{\beta }_{3}{Z}_{{ic}}+{\lambda }_{i}+{\varepsilon }_{{ic}}$$

Equation (1) was the first stage, where $D$ indicates retirement status, and ${T}_{i}=1\left({X}_{i}\ge {X}_{0}\right)$ is a dummy indicating the point of treatment discontinuity. $Z$ denotes a vector of control variables including subjective health status, age, vaccination status for other viruses, use of medicine, having cancer, and the use of the internet since the start of the pandemic. Equation (2) was the second stage, where$\,{Y}_{{ic}}$ is a variable capturing whether an individual $i$ in country $c$ was vaccinated or willing to get vaccinated. We also controlled for country dummies to capture specific country characteristics (${\lambda }_{i})$. In addition, $v$ and $\varepsilon$ are idiosyncratic error terms.

In the second equation, the effect of retirement (i.e., local average treatment effect, or the effect on compliers, i.e., the effect for those crossing the threshold and retire) on vaccination status was given by ${\beta }_{1}$. Following related literature^3,8^, we chose a window of $\pm 4$ years either side of the threshold when using the simple i model, allowing us to identify the treatment effect with sufficient precision and to consider only those individuals close to the retirement threshold. Given that the bandwidth selection involved a tradeoff between precision and bias, we also estimated our model using alternative bandwidths.

In addition, given that regression discontinuity designs tend to be sensitive to the underlying functional form, we estimated the local nonparametric model using a triangular kernel, so that observations closer to the threshold were weighted higher^22,23^. This approach was more suited to our analysis, as it was more flexible and did not rely on a specific functional form on the age-health profile^35^. We performed all further estimations using this method, which consists our baseline approach. We considered the results of robust and bias-corrected RDD as the main specification, as they tend to derive estimations that are closer to the actual treatment effect^41^. To compute the optimal bandwidths, we followed the approaches proposed by Calonico et al.^22,23,42^.

## Supplementary information

Supplementary information

## Data Availability

The datasets generated and/or analysed during the current study are available in the SHARE repository, https://share-eric.eu/data/.
