# Supplementary material for: Effect of retirement on COVID-19 vaccination in Europe: a quasi-experimental study
Source: NPJ Vaccines. 2025 Nov 20;10:241. doi: 10.1038/s41541-025-01290-y (PMC12634678; doi:10.1038/s41541-025-01290-y)
Supplement: Supplementary file 1 — Supplementary information [file 41541_2025_1290_MOESM1_ESM.pdf]

Effect of retirement on COVID-19 vaccination in Europe: a quasi-experimental study

Online Supplementary Material

Literature review

Early studies in the field of psychology and social science presented contradictory findings on the connection between retirement and health,[1–3] while more recent research has focused on addressing the potential endogeneity of retirement decisions. This is a crucial consideration from an empirical standpoint because the observed association between retirement and health may either reflect the direct impact of retirement or result from the fact that individuals in poorer health are more inclined to retire.[4] Studies of the health impacts of retirement that have adjusted for potential endogeneity have presented a mixed picture. These discrepancies can largely be attributed to variations in the countries studied and the retirement schemes in place, the specific health indicators examined, and, to a lesser extent, the methods of estimation.[5] In addition to overall uncertainty regarding the presence and direction of the retirement effect on health, there is notable heterogeneity in effects across characteristics such as gender, socioeconomic status, and occupation.[6,7]

Some studies, employing both objective and subjective health measures, have demonstrated that retirement can lead to improvements in morbidity, primarily attributed to the adoption of more health-promoting behaviors and engagement in health-enhancing activities.[8,9] This is corroborated by other studies that have associated retirement with a positive impact on five-year survival rates,[10] self-reported health status,[11] a decrease in the number of chronic conditions, and improvements in mental health and overall well-being.[12,13] Another study found that retirement positively affects physical health, with the effect being greater among individuals of low socioeconomic status.[14] Abeliansky and Strulik (2023) also show that health of those working in demanding low-status occupations is benefited from retirement.[15] In addition to its influence on individual health, research by Zang (2020) has explored potential retirement externalities, revealing positive spousal health spillovers induced by a partner's retirement.[16]

However, not all studies confirm the health-promoting effects of retirement. Some have found that retirement may lead to lower utilization of healthcare services, although statistically significant effects on physical health indicators or mortality have not been detected.[17,18] Meanwhile, using extensive United States administrative datasets, another study, observed a discontinuous increase in mortality rates following retirement, especially among males.[19] In contrast, a study by Hernaes et al. (2013) reported no significant retirement effect on mortality.[20] Other studies have demonstrated that retirement can be detrimental to health by increasing the risk of chronic disease, including cardiovascular disease and cancer, as well as negatively impacting self-rated health[6,21] and reduced mobility and daily living ability [22]. In addition, retirement has been linked to cognitive decline.[23–25] Another study also found that retirement tends to worsen health for most workers, although their findings exhibited heterogeneity based on employment type, with positive health effects identified for individuals retiring from physically demanding jobs. [26]

Research on the impact of retirement on health behaviors has also yielded mixed results. Some studies suggest that retirement can have adverse effects on health behaviors, including reduced physical exercise and increased BMI, alcohol consumption, and smoking, especially among males and those with lower levels of educational attainment.[21,27–29] Muller and Shaikh (2018) found that an individual’s retirement can have negative effects on their spouse’s alcohol consumption and physical activity.[30] While some studies have found little or no evidence for the positive impacts of retirement on health behaviors,[4,17] others have associated retirement with an increase in individual health-promoting behaviors, such as engaging in more physical exercise, reducing smoking, lowering BMI, and improving sleep duration.[8,9]

Figure S1: Share of retired individuals

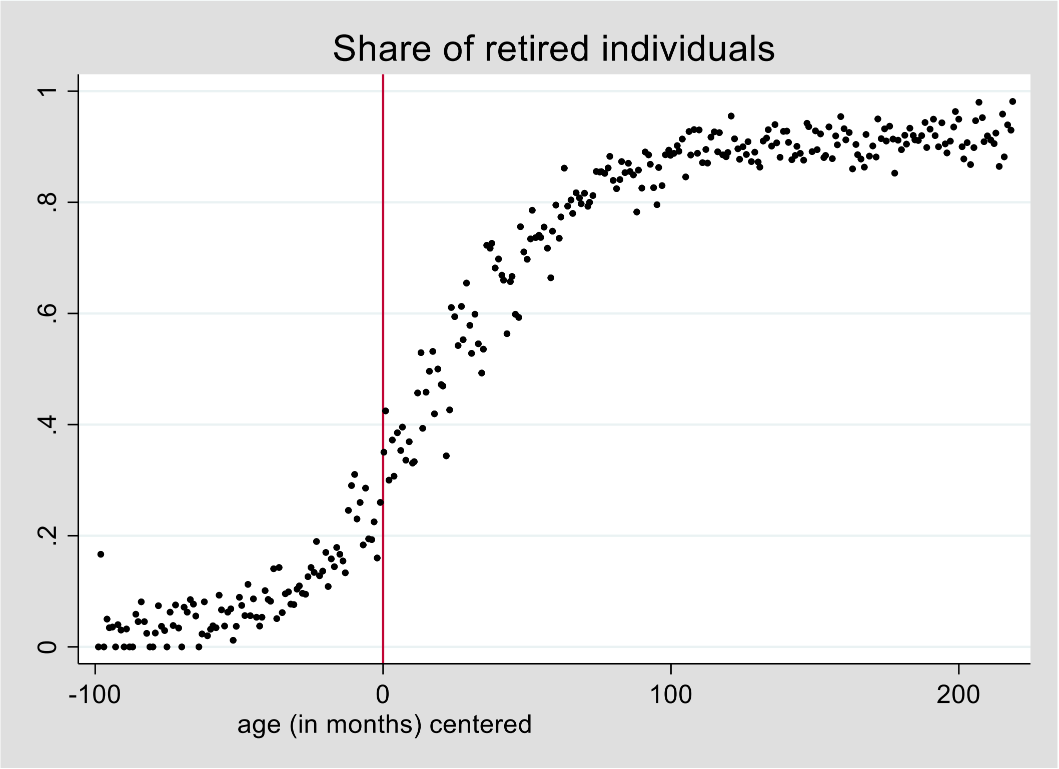

Figure S2: Retirement responses to age-based eligibility threshold

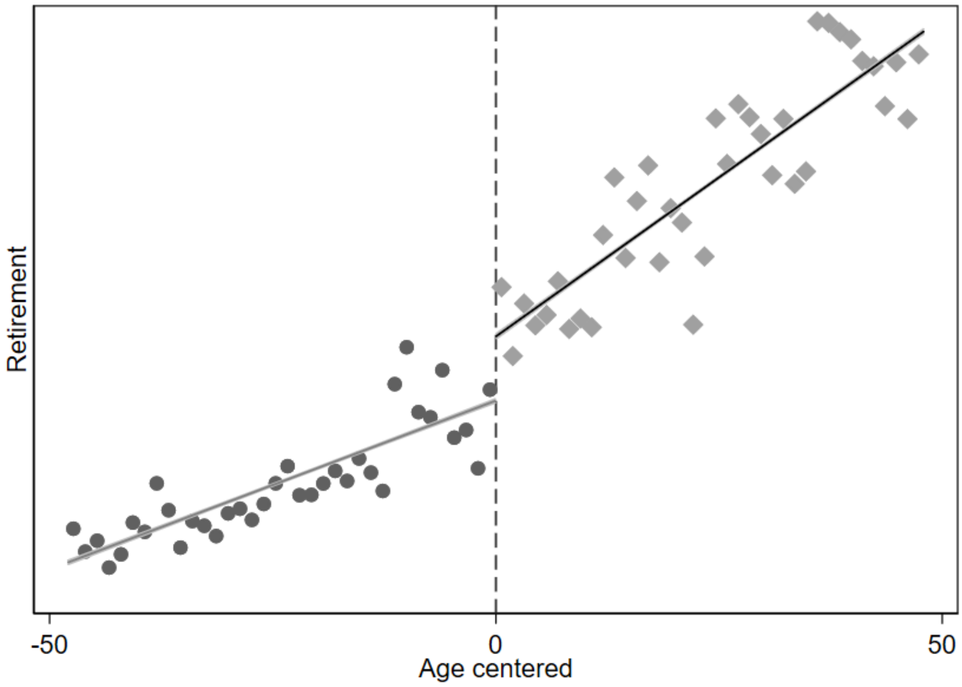

Figure S3: Discontinuity in vaccination at the age eligibility threshold

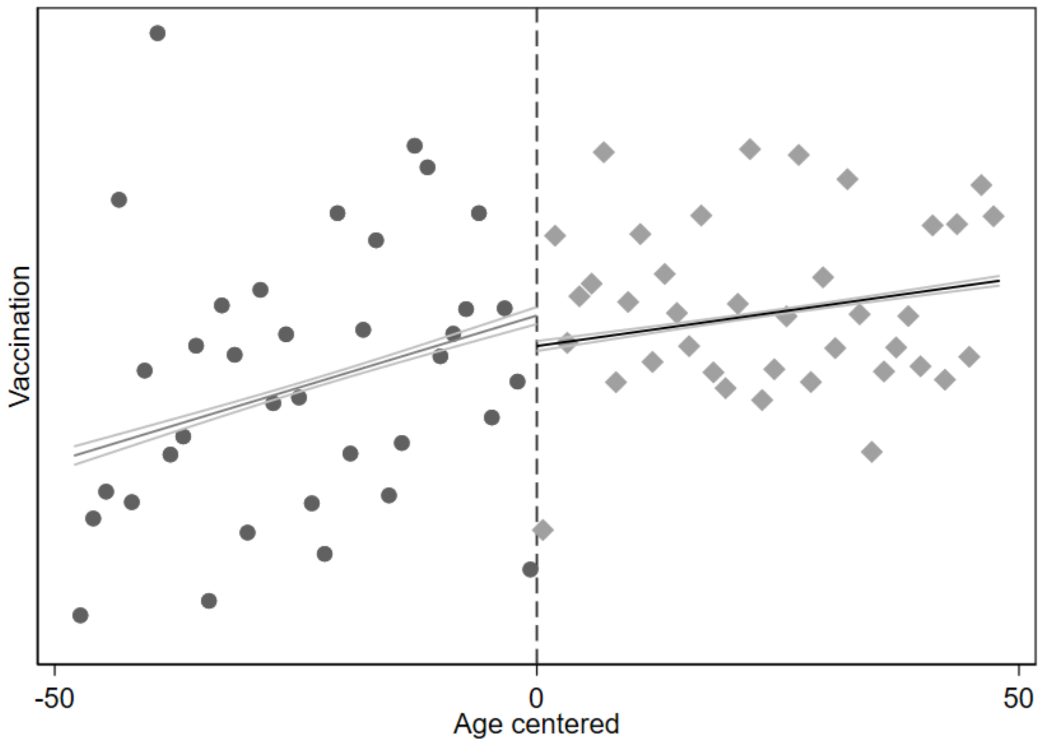

Table S1: McCrary test for RDD

Method	T Statistic	p-value	Interpretation
Robust	1.2782	0.2012	Fail to reject the null: No evidence of manipulation

Table S2: Retirement and COVID-19 vaccination (additional specifications)

	(1) Four-year bandwidth (2SLS)	(2) Three-year upper bandwidth (2SLS)	(3) Two-year upper Bandwidth (2SLS)	(4) Second Order Polynomial (2SLS)	(5) Nonparametric without covariates	(6) Nonparametric controlling for additional health conditions	(7) Nonparametric controlling for Oxford Stringency Index	(8) Only top 30% of the Oxford Stringency Index
Second Stage Estimates								
Retirement status	-0.424*	-0.440*	-0.502*	-0.423*	-0.572**	-0.718**	-0.553*	-0.383*
	(-1.84)	(-1.65)	(-1.89)	(-1.83)	(-2.14) (-1.76)	(-2.55) (-2.10)	(-1.67) (-1.48)	(-1.75) (-1.47)
First Stage								
Estimates	0.062***	0.058***	0.067***	0.063***	0.042**	0.042**	0.044**	0.061***
Eligibility for ER	(4.10)	(3.55)	(3.67)	(4.10)	(2.55)	(2.53)	(2.38)	(2.74)
Observations	11222	9515	7845	11222	11749	11612	9036	7181
Covariates	Yes	Yes	Yes	Yes	No	Yes	Yes	Yes
Country dummies	Yes	Yes	Yes	Yes	Yes	Yes	Yes	Yes
Bandwidth	48	-48, 36	-48, 24	48	50.847	50.54	38.69	56.92
F	40.98	34.16	25.91	39.98				
Polynomial Order	1	1	1	2	1	1	1	1
First Stage F	16.80	16.38	13.44	16.38				

Notes: Columns 1–3 correspond to the standard parametric 2SLS model. The covariates used are subjective health status, age, vaccination status for other viruses, use of medicine, having cancer and the use of the internet since the start of the pandemic. Column 4 presents the estimates from the baseline model after controlling only for age and country dummies. The results are local polynomial regression discontinuity (RD) estimates using the optimal bandwidth selection procedure. Two different methods (bias-corrected and robust estimator) for computing heteroskedasticity robust standard errors apply. Bias corrected t-statistics in the first parenthesis and robust t-statistic in the second. *, **, *** indicate significant at the 10%, 5%, 1% level, respectively. Column 5 presents the estimates of the baseline model, after controlling for a set of health conditions including diabetes or high blood sugar, high blood pressure or hypertension, heart attack, myocardial infarction, coronary thrombosis, or congestive heart failure, and chronic lung disease. Column 6 presents the estimates of the baseline model, after controlling for the Oxford COVID-19 Government Response Stringency Index. Column 7 presents the estimate of the baseline model focusing on the top 30% of the Oxford Stringency Index, thus capturing high-stringency settings where individuals may have perceived greater risk.

Table S3: Underlying mechanisms

	(1) Contact with family	(2) Contact with neighbours, friends, and colleagues
Second Stage Estimates		
Retirement status	1.721**	-1.380*
	(1.96) (1.70)	(-1.69) (-1.47)
First Stage		
Estimates	0.071***	0.07***
Eligibility for ER	(2.93) (2.56)	(3.49) (2.94)
Observations	6592	3495
Covariates	No	No
Country dummies	No	No
Bandwidth	41.31	40.12
Polynomial Order	1	1

Notes: All results are local polynomial regression discontinuity (RD) estimates using the optimal bandwidth selection procedure (Calonico et al. 2014, 2018). Two different methods (bias-corrected and robust estimator) in computing heteroskedasticity robust standard errors apply. Significance levels (bias corrected t statistics in the first parenthesis and robust t-statistic in the second): ***0.01, **0.05, *0.1.

Table S4: Jackknife analysis

Country Excluded	Coefficient	Country Excluded	Coefficient
Austria	-0.637 (-2.10)	Latvia	-0.788 (-2.66)
Belgium	-0.72 (-2.80)	Lithuania	-0.78 (-2.18)
Croatia	-0.68 (-2.44)	Luxembourg	-0.64 (-2.27)
Cyprus	-0.57 (-2.29)	Malta	-0.55 (-1.88)
Czech Republic	-0.63 (-2.55)	Netherlands	-0.69 (-2.17)
Denmark	-0.56 (-2.33)	Poland	-0.94 (-1.74)
Estonia	-0.51 (-2.18)	Portugal	-0.69 (-2.58)
Finland	-0.80 (-2.38)	Romania[1]	-0.49 (-1.55)
France	-0.76 (-2.89)	Slovakia	-0.56 (-2.10)
Germany	-0.82 (-2.99)	Slovenia	-0.56 (-2.14)
Greece	-0.98 (-2.93)	Spain	-0.64 (-2.06)
Israel	-0.602 (-2.11)	Sweden	-0.64 (-2.27)
Italy	-0.44 (-1.89)	Switzerland	-0.51 (-2.45)

[1] The highest difference occurs when Romania is dropped, but a standard coefficient equality test between two equations (i.e., when using the full sample versus when excluding Romania) does not reject the null hypothesis that the coefficients are equal (t statistic = -0.38). Thus, we conclude that the difference is not statistically different.

Table S5: Estimates excluding high-risk individuals

	(1) Without cancer	(2) Without chronic lung disease	(3) Without high blood pressure	(4) Without diabetes	(5) Without heart attack
Second stage Estimates					
Retirement status	-0.742*** (-2.74) (-2.26)	-0.702** (-2.30) (-1.89)	-0.879*** (-2.97) (-2.46)	-0.584* (-1.90) (-1.57)	-0.619** (-2.14) (-1.79)
First stage Estimates					
Eligibility for ER	0.041** (2.53)	0.043** (2.46)	0.030* (1.66)	0.069*** (3.44)	0.053*** (2.87)
Observations (Total)	11575	10362	8660	7782	9147
Covariates	Yes	Yes	Yes	Yes	Yes
Country dummies	Yes	Yes	Yes	Yes	Yes
Bandwidth	52.94	47.93	62.28	37.93	43.03
Polynomial Order	1	1	1	1	1

Notes: All results are local polynomial regression discontinuity (RD) estimates using the optimal bandwidth selection procedure. Two different methods (bias-corrected and robust estimator) in computing standard errors apply. Bias corrected t-statistics in the first parenthesis and robust t-statistic in the second. *, **, *** indicate significant at the 10%, 5%, 1% level, respectively.

Table S6: Additional controls

	(1)<br>Controlling for gender	(2)<br>Controlling for gender and subjective household financial situation
Second Stage Estimates		
Retirement Status	-0.633**<br>(-2.31) (-1.73)	-0.718*<br>(-1.79) (-1.60)
First stage Estimates		
Eligibility for ER	0.046**<br>(2.76)	0.042**<br>(1.92)
Observations (Total)	11222	6686
Covariates	Yes	Yes
Country dummies	Yes	Yes
Bandwidth (Right)	48.66	44.87
Polynomial Order	1	1

Notes: All results are local polynomial regression discontinuity (RD) estimates using the optimal bandwidth selection procedure (Calonico et al. 2014, 2018). Two different methods (bias-corrected and robust estimator) in computing heteroskedasticity robust standard errors apply. Significance levels (bias corrected t statistics in the first parenthesis and robust t-statistic in the second): ***0.01, **0.05, *0.1. Column 1 presents the estimates after controlling for gender and the baseline covariates. Column 2 shows the estimates after controlling for gender, subjective household financial situation and the baseline covariates.

Table S7: Effect of retirement on covariates

	(1)	(2)	(3)	(4)
	Rating of subjective health	Prescription medicines	Cancer	Use of internet since the start of the COVID-19 pandemic
Second Stage Estimates				
Retirement status	-0.579<br>(-0.88) (-0.78)<br>[-1.86 – 0.709]	0.00815<br>(0.03) (0.02)<br>[-0.587-0.603]	-0.0365<br>(-0.29) (-0.24)<br>[-0.286 – 0.213]	0.237<br>(0.86) (0.73)<br>[-0.304 – 0.779]
First Stage				
Estimates<br>Eligibility for ER	0.065***<br>(3.09)	0.061***<br>(3.09)	0.056***<br>(3.00)	0.064***<br>(3.20)
Observations	8883	9549	10617	9110
Covariates	No	No	No	No
Country dummies	No	No	No	no
Bandwidth	37.93	40.80	45.29	37.93
Polynomial Order	1	1	1	1

Notes: All results are local polynomial regression discontinuity (RD) estimates using the optimal bandwidth selection procedure (Calonico et al. 2014, 2018). Two different methods (bias-corrected and robust estimator) in computing heteroskedasticity robust standard errors apply. Significance levels (bias corrected t statistics in the first parenthesis and robust t-statistic in the second. ***0.01, **0.05, *0.1.

Table S8: Effect of own and partner's retirement on COVID-19 vaccination

	(1)<br>Four-year bandwidth<br>(2SLS)
Second Stage Estimates	
Retirement status	-0.300*
	(-1.69)
Retirement status of	-0.019
Partner	(-0.38)
First Stage	
Estimates	0.072***
Eligibility for ER (own)	(4.99)
Eligibility for ER	0.255***
(partner)	(18.05)
Observations	8162
Covariates	Yes
Country dummies	Yes
Bandwidth	48
F	30.84
Polynomial Order	1
First Stage F (own)	12.46
First Stage F (partner)	163.46

Table S9: Measurement of COVID-19 vaccination variables in SHARE

Have you been vaccinated against COVID-19?	• Yes<br>• No
Do you want to get vaccinated against COVID-19?	• Yes, I already have a vaccination scheduled<br>• Yes, I want to get vaccinated<br>• No, I do not want to get vaccinated<br>• I am still undecided

Table S10: Early retirement age thresholds

**Country**	**ERA**	**Country**	**ERA**
Austria	65 (60)	Latvia	64 (62)
Belgium	62	Lithuania	64 and 4 months (63 and 8 months)
Croatia	60 (57 and 9 months)	Luxembourg	60
Cyprus	63	Malta	61
Czech Republic	60	Netherlands	66 and 4 months
Denmark	65	Poland	66 (61)
Estonia	60	Portugal	65
Finland	63	Romania	60 (56 and 6 months)
France	61	Slovakia	60
Germany	65 (63)	Slovenia	60 (59)
Greece	62	Spain	61
Israel	67 (62)	Sweden	61
Italy	63 (62)	Switzerland	63 (62)

Note: Early retirement ages reported for men and women (in parentheses, if different).

Table S11: Heterogeneous effect of retirement on vaccination by level of social interaction

	(1)<br>Four-year bandwidth<br>(2SLS)
Second Stage Estimates	
Retirement status	-0.391*
	(-1.85)
Retirement * high	-0.104*
Contact frequency	(-1.74)
First Stage	
Estimates	0.112***
Eligibility for ER (own)	(6.82)
Eligibility * contact	-0.113***
Frequency	(-8.28)
Observations	11222
Covariates	Yes
Country dummies	Yes
Bandwidth	48
F	37.93
Polynomial Order	1

Table S12: Heterogeneity by gender

	(1)<br>Female	(2)<br>Male
Second Stage Estimates		
Retirement Status	-1.081*<br>(-1.79) (-1.45)	-1.087**<br>(-2.34) (-2.07)
First stage Estimates		
Eligibility for ER	0.032<br>(1.457)	0.051*<br>(1.92)
Observations (Total)	5867	4571
Covariates	Yes	Yes
Country dummies	Yes	Yes
Bandwidth (Right)	44.15	45.18
Polynomial Order	1	1

Notes: All results are local polynomial regression discontinuity (RD) estimates using the optimal bandwidth selection procedure (Calonico et al. 2014, 2018). Two different methods (bias-corrected and robust estimator) in computing heteroskedasticity robust standard errors apply. Significance levels (bias corrected t statistics in the first parenthesis and robust t-statistic in the second) ***0.01, **0.05, *0.1.

Table S13: Heterogeneity by countries that prioritised vaccination

	(1)<br>High priority	(2)<br>Low priority
Second Stage Estimates		
Retirement status	-0.86**	-0.56*
	(-2.00) (-1.73)	(-1.82)(-1.52)
	[-1.67, -0.016]	[-1.17, 0.042]
First Stage Estimates		
Eligibility for ER	-0.083**	0.033*
	(2.48)	(1.77)
Observations	3014	8397
Covariates	Yes	Yes
Country dummies	Yes	Yes
Bandwidth	34.967	58.731
Polynomial Order	1	1

Notes: All results are local polynomial regression discontinuity (RD) estimates using the optimal bandwidth selection procedure (Calonico et al. 2014, 2018). Two different methods (bias-corrected and robust estimator) in computing heteroskedasticity robust standard errors apply. Significance levels (bias corrected t statistics in the first parenthesis and robust t-statistic in the second) ***0.01, **0.05, *0.1.

References

1 Ekerdt DJ, Bosse R, LoCastro JS. Claims that retirement improves health. *J Gerontol* 1983; **38**: 231–6.

2 Legacy AU, Minkler M. Research on the Health Effects of Retirement: An Uncertain Legacy. *J Health Soc Behav* 1981; **22**: 117–30.

3 Moen P. A life course perspective on retirement, gender, and well-being. *J Occup Health Psychol* 1996; **1**: 131–44.

4 Rose L. Retirement and health: Evidence from England. *J Health Econ* 2020; **73**: 102352.

5 Filomena M, Picchio M. Retirement and health outcomes in a meta-analytical framework. *J Econ Surv* 2022. DOI:10.1111/JOES.12527.

6 Ebeid M, Oguzoglu U. Short-term effect of retirement on health: Evidence from nonparametric fuzzy regression discontinuity design. *Health Econ* 2023. DOI:10.1002/HEC.4669.

7 Grøtting MW, Lillebø OS. Health effects of retirement: evidence from survey and register data. *J Popul Econ* 2020; **33**: 671–704.

8 Insler M. The Health Consequences of Retirement. *Journal of Human Resources* 2014; **49**: 195–233.

9 Eibich P. Understanding the effect of retirement on health: Mechanisms and heterogeneity. *J Health Econ* 2015; **43**: 1–12.

10 Bloemen H, Hochguertel S, Zweerink J. The causal effect of retirement on mortality: Evidence from targeted incentives to retire early. *Health Econ* 2017; **26**: e204–18.

11 Coe NB, Zamarro G. Retirement effects on health in Europe. *J Health Econ* 2011; **30**: 77–86.

12 Gorry A, Gorry D, Slavov SN. Does retirement improve health and life satisfaction? *Health Econ* 2018; **27**: 2067–86.

13 Latif E. The impact of retirement on psychological well-being in Canada. *J Socio Econ* 2011; **40**: 373–80.

14 Grøtting MW, Lillebø OS. Health effects of retirement: evidence from survey and register data. *J Popul Econ* 2020; **33**: 671–704.

15 Abeliansky AL, Strulik H. Health and aging before and after retirement. *J Popul Econ* 2023; **36**: 2825–55.

16 Zang E. Spillover effects of a husband's retirement on a woman's health: Evidence from urban China. *Soc Sci Med* 2020; **245**. DOI:10.1016/J.SOCSCIMED.2019.112684.

17 Johnston DW, Lee WS. Retiring to the good life? The short-term effects of retirement on health. *Econ Lett* 2009; **103**: 8–11.

18 Nielsen NF. Sick of retirement? *J Health Econ* 2019; **65**: 133–52.

19 Fitzpatrick MD, Moore TJ. The mortality effects of retirement: Evidence from Social Security eligibility at age 62. *J Public Econ* 2018; **157**: 121–37.

20 Hernaes E, Markussen S, Piggott J, Vestad OL. Does retirement age impact mortality? *J Health Econ* 2013; **32**: 586–98.

21 Behncke S. Does retirement trigger ill health? *Health Econ* 2012; **21**: 282–300.

22 Dave S, Rashad I, Spasojevic J. The Effects of Retirement on Physical and Mental Health Outcomes. *South Econ J* 2008; **75**: 497–523.

23 Mazzonna F, Peracchi F. Ageing, cognitive abilities and retirement. *Eur Econ Rev* 2012; **56**: 691–710.

24 Bonsang E, Adam S, Perelman S. Does retirement affect cognitive functioning? *J Health Econ* 2012; **31**: 490–501.

25 Rohwedder S, Willis RJ. Mental Retirement. *Journal of Economic Perspectives* 2010; **24**: 138.

26 Mazzonna F, Peracchi F. Unhealthy Retirement? *Journal of Human Resources* 2017; **52**: 128–51.

27 Ayyagari P. The Impact of Retirement on Smoking Behavior. *East Econ J* 2016; **42**: 270–87.

28 Feng J, Li Q, Smith JP. Retirement Effect on Health Status and Health Behaviors in Urban China. *World Dev* 2020; **126**. DOI:10.1016/J.WORLDDEV.2019.104702.

29 Godard M. Gaining weight through retirement? Results from the SHARE survey. *J Health Econ* 2016; **45**: 27–46.

30 Müller T, Shaikh M. Your retirement and my health behavior: Evidence on retirement externalities from a fuzzy regression discontinuity design. *J Health Econ* 2018; **57**: 45–59.
